# Importance of Nano Medicine and New Drug Therapies for Cancer

**DOI:** 10.34172/apb.2021.052

**Published:** 2020-10-19

**Authors:** Arash Abdolmaleki, Asadollah Asadi, Krishnamoorthy Gurushankar, Tahereh Karimi Shayan, Fatemeh Abedi Sarvestani

**Affiliations:** ^1^Department of Engineering Sciences, Faculty of Advanced Technologies, University of Mohaghegh Ardabili, Namin, Iran.; ^2^Bio Science and Biotechnology Research center (BBRC), Sabalan University of Advanced Technologies (SUAT), Namin, Iran.; ^3^Department of Biology, Faculty of Science, University of Mohaghegh Ardabili, Ardabil, Iran.; ^4^Laboratory of Computational Modeling of Drugs, Higher Medical and Biological School, South Ural State University, Chelyabinsk-454 080, Russia.; ^5^Department of Physics, Kalasalingam Academy of Research and Education, Krishnankoil- 626126, Tamil Nadu, India.

**Keywords:** Cancer, Drug, Nanomedicine, Drug delivery system

## Abstract

Cancer is one of the deadly diseases leading to approximately 7.6 million deaths worldwide, with the mortality rate of 13%, and the number of deaths is expected to increase to 13.1 million within the next 10 years. In controlled drug delivery systems (DDS), the drug is transported to the desired location. Thus, the influence of drugs on vital tissues and undesirable side effects can be minimised. Additionally, DDS protects the drug from rapid degradation or clearance and enhances drug concentration in target tissues, and therefore, minimise the required dose of drug. This modern form of therapy is particularly important when there is a discrepancy between the dose and concentration of a drug. Cell-specific targeting can be achieved by attaching drugs to individually designed carriers. Recent developments in nanotechnology have shown that nanoparticles (particles with diameter < 100 nm in at least one dimension) have great potential as drug carriers. Because of their small size, these nanostructures exhibit unique physicochemical and biological properties that make them a favourable material for biomedical applications. Therefore, in this review, we aimed to describe the importance and types of nanomedicines and efficient ways in which new drug delivery systems for the treatment of cancer can be developed.

## Introduction

### Early detection

For cancer therapy, there is a constant requirement for novel technologies. Studies have reported that the number of incidences, prevalence, and mortality rate of cancer are still extremely high.^1,2^ As per the World Health Organization (WHO), cancer is one of the deadly diseases leading to approximately 7.6 million deaths worldwide, that is, the mortality rate being 13%. The deaths are expected to increase to 13.1 million within the next 10 years. Cancer is considered as a combination of several diseases with a distinct set of diseases produced in individual organs or through distinct mechanism. Many factors potentially leading to cancer can be prevented because several reports suggest that detrimental behavioural patterns such as smoking and unhealthy dietary habits lead to 30% of the deaths by cancer.^3,4^ However, through simple behavioural changes, most cancers cannot be prevented and need technological innovation to improve results. Surgery, radiation, and chemotherapy are essential lines of treatment for cancer. However, these modern treatment lines have severe side effects. Despite advances in the treatment strategies for cancer, most practical methods aimed at ensuring safety and improving the life quality of patients with cancer still require improvement in terms of early detection and identification of curable precursors of cancers. The lack of such treatment has led to increased interest in using early detection as a potential approach to manage cancer progression. Early detection of abnormal tissue or cancer is important because of the ease of treatment at early stages, that is, early detection is important because if an abnormal tissue or cancer is detected at an early stage, the treatment is easier. Early detection of cancer can profoundly enhance recovery rates; it can be achieved by promotion of early diagnosis through education and screening methods. Identification of potential warning signs of cancer and further investigations help in early diagnosis. Boosting awareness amongst doctors, other healthcare staff, and general public over possible cancer warning signs can help significantly. Cancer diagnosis at the early phase of carcinogenesis is a critical step in cancer treatment. A significant success has been achieved in reducing cancer caused by viruses, such as human papillomavirus.^5,6^ This aspect can be progressed through comprehensive adoption of vaccination, in addition to the use of nanotechnology and other technologies to improve vaccine efficiency.^5,7^ The tumour should be identified at its initial steps; otherwise the treatment involves strategies that uproot the cells of fully developed cancers keeping the healthy cells intact. Targeted cancer therapy uses target-specific drugs that invade cancer cells and block the growth and metastasis of cancer cells by interfering with specific molecules involved in carcinogenesis and tumour growth. To overcome the disadvantages of current cancer treatment techniques, scientific community has turned to nanotechnology to develop newer and more effective drug carrier systems to safely deliver anticancer drugs to cancer cells. Research in nanomedicine and nanotechnology has encouraged the development of innovative treatment methods on nano scale because of their low toxic side effects, small size, and controlled drug release. Nanomedicine involves research and development of nano-scale technology, tools, and drug delivery system for preventing, identifying, and treating diseases. The incidences of early diagnosis of cancer can be increased using nanotechnology through enhanced images; this can lead to better results associated with the more efficient deployment of new screening technologies.^8^ When drugs are loaded onto nanoparticles (NPs) through physical encapsulation, adsorption, or chemical conjugation, the pharmacokinetics and therapeutic index of the drugs can be significantly improved as compared with the free drug counterparts. Nanostructured biomaterials and NPs have unique physicochemical properties such as ultra-small and controllable size, large surface area to mass ratio, high reactivity, and functionalisable structure. Particle size and size distribution are some of the most widely accepted defining characteristics of nanoparticle-based medicines because size can significantly impact the pharmacokinetics, biodistribution, and safety. The size of NPs used in a drug delivery system should be large enough to prevent their rapid leakage into blood capillaries but small enough to escape capture by macrophages that are lodged in the reticuloendothelial system, such as the liver and spleen. Nanostructures, as described, are geometric configurations with sizes ranging from 1 to 100 nm. Nevertheless, with the development of nanostructures, their range of applications increased.^9^

## Technical evaluation

### Significance of nanomedicine against cancer

Different sized NPs have different biomedical uses. Biomedical application of nanosystems has been highly investigated and has shown widespread potential in various fields, particularly in cancer detection, imaging, and therapy.^10,11^ Nanotechnology is the science and technology that deals with precisely manipulating the structure of a material at the molecular level. It is a small scale of material manipulation and application. Molecules and atoms function differently when they have small sizes, leading to various interesting applications. The term cancer nanotechnology was acknowledged by the National Cancer Institute, which considers that nanotechnology offers an extraordinary, paradigm-shifting opportunity to make significant advances in cancer diagnosis and treatment.^12^ NPs, as individual solid particles or dispersions, have size between 10 and 100 nm. Polymeric magnetic and micelles NPs, NPs of colloidal gold, and NPs of ceramic are under investigation to be adopted in drug delivery systems.^13^ For localisation in tumour cells, these nanoparticle-based drug delivery systems can be coated with tumour-specific antibodies, peptides, sugars, hormones and anti-carcinogenic drugs. These NPs have been effectively coupled with the aforementioned anti-carcinogenic chemotherapeutic or chemopreventive agents and have been tested for their target specificity.^12^ Such NPs are far better than traditional methods of drug delivery because they have nano-scale receptors on their surface. Chemotherapeutic agents can be characterised for certain organs in body.^12^

## Advantages of NPs

**Figure 1:**
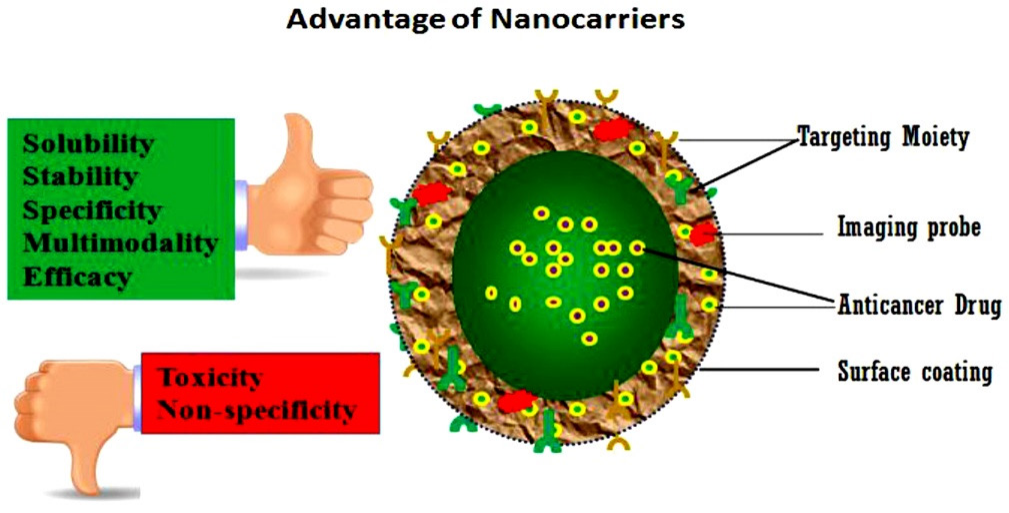
Advantage of nanomaterials. Nanomaterials as nano-carriers can improve solubility, stabilization, specificity, multimodality and efficiency, whereas decreasing toxic side effects also enhancing the non-specificity of traditional cancer therapies.

Drugs can be protected from getting degraded using capsules coat made up of NPs. Because NPs are very tiny, they can easily break into smaller capillaries and absorb by cancer cells. This allows the target site to absorb drugs effectively. Another advantage of the nano-scale system is that they can efficiently overcome the clearance by the kidney, and therefore, provide good blood circulation time to the drugs they carry. Apart from these advantages, the most effective property of this system is the capability to provide high therapeutic potential of NPs. To provide NPs with a formulation similar to carriers of anti-carcinogenic agents, numerous approaches based on nanobiotechnology are still under development. The advantages of applying NPs as a drug delivery system include controlled and sustainable delivery of drugs that, altered distribution of drugs in organs and eventual clearance to achieve increased therapeutic effectiveness, and decreased side effects (Figure 1).^13^

## Importance of NPs in drug delivery

Drugs should be added to drug delivery systems without any chemical reaction; this is a crucial factor essential for conserving the function of drugs. In case of NPs as a drug delivery system, controlled release and degradation properties of a drug can be easily modified. For a long time, there is no wastage of drugs; therefore, bioavailability of drugs at specific sites can be increased in the right proportion. It increases the solubility of the drugs that are poorly water-soluble and extends the half-life of systemic circulation of the drug through decreasing immunogenicity. Additionally, it leads to drug release at a stable rate and reduces the duration of administration.^14,15^

## Polymer NPs

In recent years, polymeric nano-sized carriers have shown a significant potential as a drug delivery system for tumours, and nano-sized drug carriers have been minimally detected at typical tissue places, resulting in high anti-tumour therapeutic efficiency. Desirable accumulation of nano-sized polymer carrier and drug at tumour sites is described by the effect of enhanced permeability and retention (EPR) resulted from the disorganization of tumour and faulty vascular architecture.^16,17^

## Routes of administration

Drug is administered through two routes: (i) oral and (ii) intravenous (IV). IV administration allows concentrating NPs at the target areas, rerouting drugs away from the sites where they cause toxicity, and increasing the circulation time of drugs having short circulation times. IV administration of chemotherapeutics is the main source of pain, stress, and high costs because of several hospitalization events required to complete multiple sessions of IV chemotherapeutic regimens. In recent years, research on NPs as oral drug delivery vehicles has been extensively undertaken. Oral delivery of drugs using NPs has shown to be far superior to the delivery of free drugs in terms of bioavailability, retention time, and biodistribution. Oral administration of drugs is the most efficient way, but this causes many obstructions to the usage of colloidal transporters because of the extreme conditions in gastrointestinal tract that releases the drug. On the other hand, NPs could be adopted to generate a labile drug in the gastrointestinal tract by degradation or to cover the drug to protect other healthy tissues from toxicity of the drug. Because of their bioadhesive features, polymeric NPs could be fixed in mucous, and they exhibit a slower gastrointestinal clearance.^16^ The development of appropriate and efficient oral therapeutic agents could substantially contribute to patients’ life quality and greatly decrease costs, and such agents could be more effective than conventional treatment methods. There are numerous nano-based drug delivery systems: lipid-based (liposomes and lipid NPs with a solid matrix), cyclodextrin-based, and polymer-based (polymeric NPs) nanocarriers. These are considered to be among the most suitable systems for oral delivery. Various techniques, such as complexation with cyclodextrin and liposomes, have been used to increase the solubility of drug. However, cyclodextrin use is associated with a risk of nephrotoxicity, and liposomes are not stable during long-term storage. NPs have been adopted as the carriers of oral drugs for several purposes, such as increasing the bioavailability of drugs and extending the retention time of drugs having poor absorption in the intestine and increasing absorption to facilitate enhanced dispersion at molecular level.^18-20^

One of the most important potential advantages of nanotechnology for cancer treatment is tumour targeting. During cancer treatment, a precise concentration of a therapeutic agent must reach specifically to the tumour tissue after passing through diverse biological barriers present in the body. Once at the target tissue, the anti-cancer drug should have the capacity to selectively destroy cancer cells, sparing the healthy ones. Therefore, the intracellular concentration of the drug will be increased and adverse side effects and toxicity will be decreased, leading to improvement in patient compliance, quality of life, and survival. To achieve these goals efficiently, three mechanisms of tumour targeting by drugs have been described. The ability to differentiate non-malignant cells from malignant cells and selectively eliminate malignant cells is one of the main tasks of nanotechnology because of its association with cancer treatment.^21,22^

## Passive targeting through EPR

EPR is well known to have leakage of tumour vessels compared with the hierarchical structure of natural vessels, partly because malignant cells have no response to the signal from the cell needed for vasculogenesis in order.^23^ The tumour can be reached by macromolecules through leaky vasculature and survive in part due to decreased clearance of lymph^24^ in tumours through a process called EPR.^25,26^ In tumour tissues through EPR, several NPs are found, comprising of multi-walled and single-walled carbon nanotube,^27^ and liposomes, and viral NPs^28^ that are substantially different with respect to density and 100s of nm with a single dimension of other globular proteins and NPs features were documented to locate the tissue of tumour through the EPR. To the best of our knowledge, there was no systematic investigation into the related efficiency of the position of the tumour through the EPR for specific NPs in tumour models. Recently, an interesting form of passive targeting through EPR has been explained, in which gold nanorods were delivered through EPR to tumour tissues and were adopted to heat and melt the tumour after laser irradiation.^29-31^

## pH-activated NPs for cancer treatment

**Figure 2:**
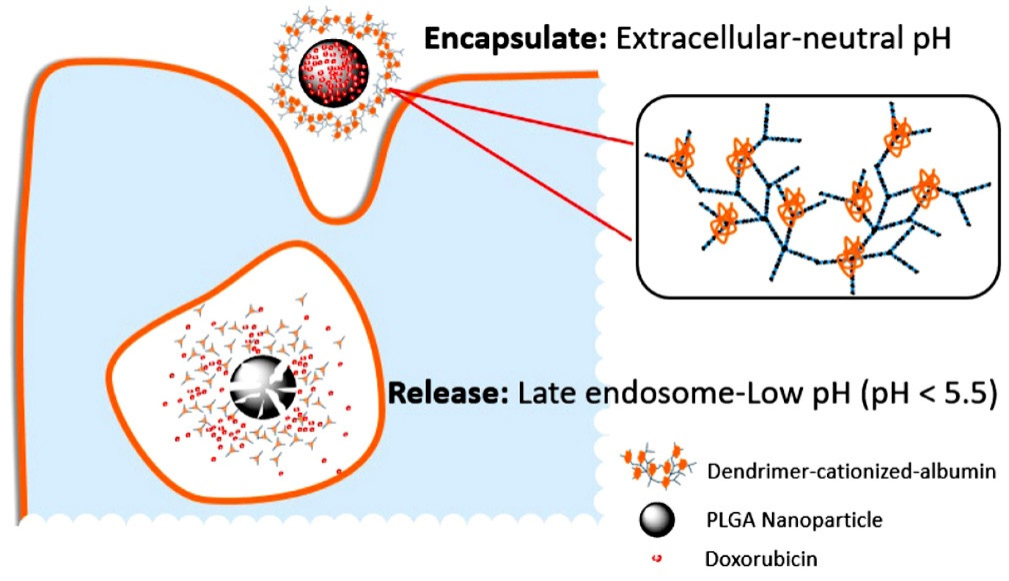
Figure shows the graphic description of the dissolution of poly(lactic-co-glycolic-acid) (PLGA) covered dendrimer-cationised-albumin nanoparticles. According to the acidic late endosomal compartment pH, doxorubicin (DOX) is released into U-87 MG glioblastoma cells.

Because healthy and cancerous cells have different pH, pH-based NPs were developed for cancer treatments which release the drugs depending on the pH of the cells. At specific places in the body, the drug release could be activated as the response to physiological pH changes.^32^ However, because of varied pH values, oral administration is particularly tricky, and most oral therapeutics need controlled protection or release; retention time of orally administered drugs is expected to decrease because of their denaturation in the stomach due to the highly acidic environment.^33^ It can result in less drug concentration at the target spot, leading to multi-drug resistance.^34^ However, the mechanism of intestinal release directly triggered by pH can be advantageous. On the other hand, in the presence of various enzymes such as pepsin and pancreatic enzymes, solid lipid NPs are formed which release drugs only at the gut pH. Additionally, various polymeric materials such as poly(lactic-co-glycolic-acid) (PLGA)^35^ and polyacrylic acid^36^ were described to produce adequate levels of anti-tumour agents through oral administration.^36^ Furthermore, oral systems were developed to deliver natural products, such as curcumin, which for many years, was assumed to have anti-cancer properties. Insolubility of curcumin in water (at ng level) greatly limits its oral administration.^37^ Cui et al showed that a new microemulsifying drug delivery system can greatly increase solubility of curcumin in water and the adsorption rate by pH-responsive release in the gastrointestinal tract. The rate of adsorption was increased to >90% within 12 h as compared with only one-fifth of the free curcumin.^38^ As mentioned earlier, tumour tissues may have a much lower microenvironmental pH than the healthy tissues.^39-41^ In cancer cells, pH of the cytoplasm is more than that in healthy cells. Lysosomal compartments act differently in the cell. In healthy cells, an acidic environment is maintained in the lysosomes, with pH ranging between 4.5 and 6.5, whereas in malignant cells, the pH of lysosomes is lower than 4.5. The pH of lysosomes may be in the range of 3.8–4.7 in highly metabolic cells.^42^ The difference between pH of lysosomes in healthy and malignant cells makes them a desirable target for receptive drug release.^43,44^ Additionally, Muniswamy et al reported a drug delivery system with a centre of doxorubicin (DOX)-loaded PLGA NPs and an outer layer of dendrimer-cationised albumin. PLGA is degraded in malignant cells at pH from 4.5 to 5.5, and release the DOX (Figure 2). Furthermore, these drug delivery systems can pass through the blood–brain barrier and blood–tumour barrier. A study on glioblastoma cells (U-87 MG) revealed that tumour cell death increased by 5.5 times due to an increase in the expression of caspase-3 gene mediating cell apoptosis.^45^ NPs enzyme activation in the body, more than 5000 biochemical processes are catalysed, which is substantially greater than those in normal tissues.^46^ This makes it possible for enzymes to directly activate the NP system at the tumour spot.^46,47^ For enzymatic activation systems, a variety of cellular enzymes have been used, including cathepsins, matrix metalloproteinases (MMPs),^48,49^ protein tyrosine kinase-7 (PTK-7), and telomerase.^46,50^

## Cathepsins

The rate of expression of cathepsins was suggested to be associated with metastasis and invasiveness in case of different types of cancer including ovarian, breast, and pancreatic cancers.^51^ Lysosomal cathepsin B can directly degrade polymers, including poly-L-lysine hydrobromide (PLL). Villar-Alvarez et al showed PLL particle coating and incorporated gold nano-rods and DOX (a chemotherapeutic agent). The assembled system could reduce the cell number of MDA-MB-231 and HeLa cells to approximately one-fifth of the original when incubated with 2, 5, and 10 NPs/mL; less than 50% of cancer cells were killed by the same concentration of only DOX.^52,53^

## MMPs

MMPs have an important role in development and progression of cancer, including metastasis and invasion. There are 23 types of MMPs in humans, numbered and characterised by collagen, gelatin, and extracellular proteins to certain substrates and cell locations based on their specificity.^46,48,54^ Both MMP-2 and MMP-14 were demonstrated as potential active biomarkers in nanosystems in the treatment of cancer.^55^ MMP-9 dissolves coating layer of polyvinylpyrrolidone (PVP) or gelatin, which can lead to release of tumour-sensitive drug. It was integrated into a proof-of-concept device that the NPs of mesoporous silica were filled by dying molecules and surface was coated with PVP. The coloured molecules were released after the PVP coating was enzymatically degraded. The signal was analysed using multispectral optoacoustic tomography, and it was discovered that it was 10 times greater in the control group treated with MMP-9.^48^

## Glycosyl hydrolases

Glycosyl hydrolase is a group of intracellular enzymes that catalyse the hydrolysis of glycosidic bonds in complex sugars and is a controlled release activator. Dzamukova et al proposed an elegant system in which a dextrin cap can be broken by glycosyl hydrolases for encapsulation of a cytotoxic drug by dextrin as a nano-terminus of nano-structure of the tubular clay has been used. As a result, drug in the existence of a great amount of glycosyl hydrolases in the tissue of cancer.^56^

## Protein tyrosine kinases

PTK-7 is one of the subgroups of kinase lacking catalysing activity but retaining roles in signal transduction. Some specific types of cancer, such as human oesophageal squamous cell carcinoma and T-cell acute lymphocytic leukaemia, were shown to have much higher expression levels of PTK-7. Although the role of PTK-7 has not been comprehensively explored, it is identified as being associated with the progression of cancer.^57^ In 2014, Huang et al. reported the hybrid of a nanoparticulated aptamer-lipid-PLGA that can co-deliver paclitaxel and DOX. The selected aptamers in this system interact with PTK-7 expressed on the cell membrane of tumour cells. When the aptamer interacts with PTK-7, the structure of aptamer changes and DOX is released which is bound to the hairpin structure of the aptamer.^58^

## Nicotinamide adenine dinucleotide phosphate dehydrogenases

Though the family of nicotinamide adenine dinucleotide phosphatide hydrogenases (NADPH) consists of several members while there are many members of the family of NADPH dehydrogenase, expression levels of NADPH are approximately 12–50 times higher in malignant cells.^59^ On this basis, a new theranostic nanoprobe called ‘Prodrug 1’ has been reported by Shin et al. The effect of NQO1 on the toxic combination 7-ethyl-10-hydroxycamptothecin (SN-38), a famous chemotherapy medication, decomposes Prodrug 1.^59^

## Telomerases

**Figure 3:**
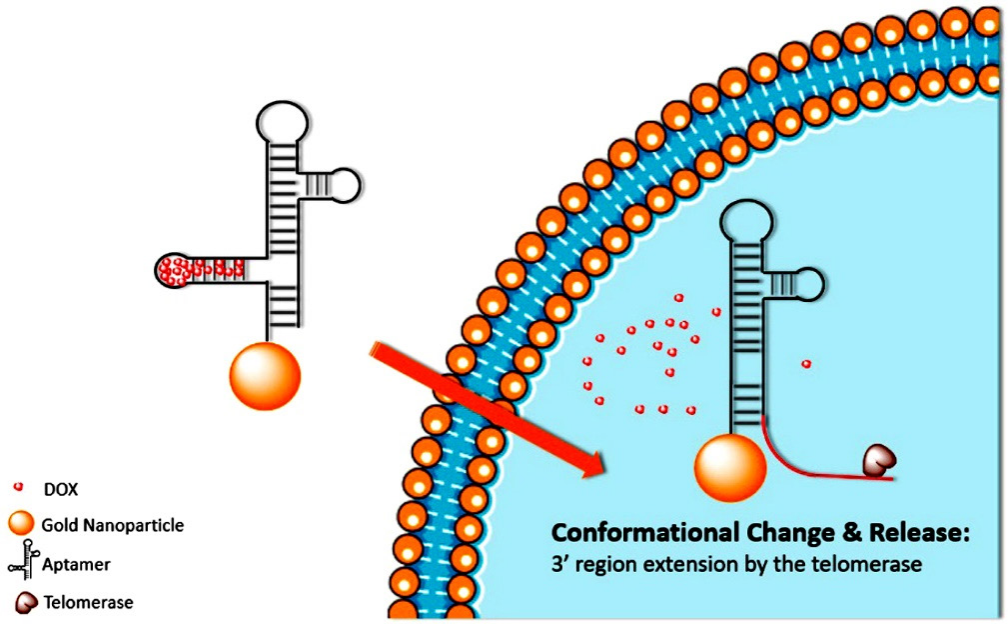
Figure shows the graphical representation of telomerase stretching the 3'endش aptamer that results in the morphology of the aptamer and the release of the DOX within tumor cells.

Shi et al proposed a single release of DOX theranostic nanoprobe activated through telomerase.^50^ The production of telomerase in malignant cells is much higher than that in healthy cells. Therefore, a new conformation of DNA shell, a hairpin, has been designed that includes 3’ telomerase primers that could be elongated by the current telomerase (Figure 3). Though the activity of telomerase in malignant cells is much higher than that in healthy cells, the elongation of 3’ regions could led to deconstruction of aptamer structure and could result in the release of the anti-cancer drug DOX and fluorescent carboxyfluorescein label within the aptamer. Using MTT assay, the nanoprobe is reported to reduce HeLa cells growth by 50% compared with almost no decline in viability of L-02 cells (control).^50^

## Enzyme-loaded NPs

Additionally, it is liable to design systems carrying enzymes to the body in addition to the use of those enzymes that already exist in cells. For example, Yang et al used a nanosystem based on the organic silica carrying glucose oxidase (GOx) and hypoxia-activated prodrug (AQ4N). The high levels of glutathione in malignant tissues lead to the degradation of organic silica and AQ4N and GOx were released. The catalysis of glucose oxidation by GOx results in an environment of hypoxic tissue allowing the non-toxic AQ4N to be turned into the cytotoxic AQ4.^60^

## Membrane proteins

Porphyrin is a component in the biosynthesis pathway of proto-haeme of haeme derived from intermediate porphyrinogens, and it naturally occurs in very low amount in living cells. During normal conditions, the protoporphyrin synthesis is regulated by feedback mechanism; in other words, cells generate protoporphyrin at a rate matching with the rate of haeme generation. However, the feedback mechanism loses control with excessive cell proliferation, and the excessive amount of porphyrin therefore produced appears in the blood and tissues. Because of the high proliferation rate, there is an intense need for iron in malignant cells, for example, in melanoma,^61^ carcinoma, and glioblastomas.^62^ Transferrins are glycoproteins that control the free iron level and help in iron binding in biological fluids. Through binding of transferrin to transferrin receptors on cell surface, iron ions could be carried into cells.^63^ Because of requirement of high iron levels in malignant cells, transferrin receptors could be adopted for tumour targeting.^64,65^

## Conclusion

In this review, we aimed to describe the importance and types of nanomedicines and efficient ways in which new drug delivery systems for the treatment of cancer can be developed.

Nanoparticles drug delivery systems are specifically proposed as an option to preserve the efficacy of newly produced, effective and complex drugs. New controlled drug delivery systems can be effective for the treatment of cancer by protecting the drugs from degradation, minimise the required dose of drug and increasing the concentration of drug in target tissues.

Although cancer is a more complicated disease than heart disease, changes in lifestyle (cessation of smoking) and availability of new drugs with advances in nanotechnology and other fields of medicine can decrease the mortality rate of cancer in the following 10 years. Thus, further studies are essential to evaluate the nanoparticles drug delivery systems for the treatment of cancer.

## Ethical Issues

Not applicable.

## Conflict of Interest

Authors declare no conflict of interest in this study.
